# Gestational Age Alters Assessment of Neonatal Abstinence Syndrome

**DOI:** 10.3390/pediatric14010009

**Published:** 2022-01-28

**Authors:** Sasha Amiri, Jayasree Nair

**Affiliations:** Department of Pediatrics, Jacobs School of Medicine and Biomedical Sciences, University at Buffalo, New York, NY 14203, USA; jnair@upa.chob.edu

**Keywords:** neonatal abstinence syndrome, neonatal opioid withdrawal syndrome, modified Finnegan scoring, prematurity, gestational age

## Abstract

Neonatal abstinence syndrome (NAS) due to maternal opioid use affects both term and preterm infants; however, the relationship between gestational age and clinical symptomatology is still poorly understood. In this study, we compared the clinical features and outcomes of NAS in infants admitted to a neonatal intensive care unit (NICU) based on gestational age groups: preterm (32–36 6/7 weeks) and term (37 weeks or older). A retrospective data analysis was conducted using the medical records of infants with a diagnosis of NAS admitted to a regional perinatal center between 2014 and 2020. A modified Finnegan scoring system was used based on three different symptom categories, including Central Nervous System (CNS), Gastrointestinal (GI) and Other. In total, 166 infants with a diagnosis of NAS were included, with 52 (31%) who were preterm and 114 (69%) who were term. The highest NAS score was significantly lower for the preterm group than for the term group. Preterm infants were less likely to require first-line pharmacotherapy with morphine (52% versus 75%) and to experience GI symptoms during their hospitalization. Newer NAS assessment modalities, such as eat, sleep, console (ESC), may overcome the existing challenges of traditional scoring systems, but will require validation in preterm infants.

## 1. Introduction

Neonatal abstinence syndrome (NAS) due to maternal opioid use affects both term and preterm infants; however, the relationship between gestational age and clinical symptomatology is still poorly understood [1,2]. The number of infants born with NAS in the United States has increased in incidence by six-fold (1.2 to 6.7/1000 births) from 2000 to 2016 [3]. The modified Finnegan scoring (MFS) tool continues to be the most commonly used method for determining the severity of and medical management for NAS [4], despite not being validated in preterm infants. 

NAS, or NOWS (neonatal opioid withdrawal syndrome), is a multisystemic disorder in newborns caused by the abrupt cessation of exposure to maternal substances in utero. It typically impacts the Central Nervous System (CNS), Gastrointestinal (GI) system and autonomic systems, resulting in clinical signs, such as irritability, poor feeding/suck, yawning, sneezing, tachypnea, fever, sweating, diarrhea, vomiting, tremors and convulsions. The type of substances most often implicated in NAS are opioids, but nicotine, anti-depressants, benzodiazepines, alcohol, methamphetamine and inhalants have also been shown to play a role in the disease process [5].

Previous studies have suggested that earlier gestational ages are associated with a lower likelihood of requiring pharmacological intervention for NAS [5,6,7]. This finding, however, is inconclusive, as some studies demonstrated only a shorter duration of pharmacotherapy in late preterm infants [1], or no relation at all [8]. In comparison, breastmilk has been widely cited as improving outcomes in NAS regardless of gestational age [9,10].

Much of the current available literature on NAS (or the newer “neonatal opioid withdrawal syndrome”) continues to treat preterm and term infants as one homogenous population [11]. Prior work by Alloco et al. hinted that NAS in premature infants can manifest differently when compared to term infants. Similarly, we hypothesized that preterm infants with NAS would have a different symptomatology than term infants, and that this would lead to reduced scoring with the MFS tool. A secondary focus of our study was to compare the NAS outcomes of infants with and without breastmilk in their diet.

## 2. Materials and Methods

### 2.1. Study Design

A retrospective data analysis was conducted using the medical records of infants admitted to the neonatal intensive care unit of John R. Oishei Children’s Hospital, a regional perinatal center, between 2014 and 2020. Institutional review board approval and waiver for use of individually identifiable health information was obtained prior to the initiation of data collection. Two queries were performed using our inclusion/exclusion criteria, outlined below, yielding 52 preterm and 114 term infant subjects. Preterm and term populations were defined by gestational ages of 32–36 6/7 weeks and ≥37 weeks respectively.

Data were collected using electronic medical records and stored in a password protected Excel spreadsheet, only accessible by the study team on password protected computers. Patient and maternal characteristics obtained by our study team included: gestational age (weeks.days/7), birth weight (grams), small for gestational age (SGA) or intrauterine growth restriction (IUGR) status, patient respiratory conditions, patient non-respiratory conditions, maternal drug exposure and maternal drug dose. Additionally recorded was the presence of polypharmacy, benzodiazepine use, selective serotonin reuptake inhibitor (SSRI) or serotonin-norepinephrine reuptake inhibitor (SNRI) use, tobacco use and alcohol use. Maternal drug exposures of importance to our study involved opiates (buprenorphine or Subutex, buprenorphine/naloxone or Suboxone, methadone, heroin, morphine, codeine, fentanyl), stimulants (amphetamine, methamphetamine, cocaine), depressants (barbiturates, benzodiazepines, alcohol) and cannabinoids.

**Figure 1:**
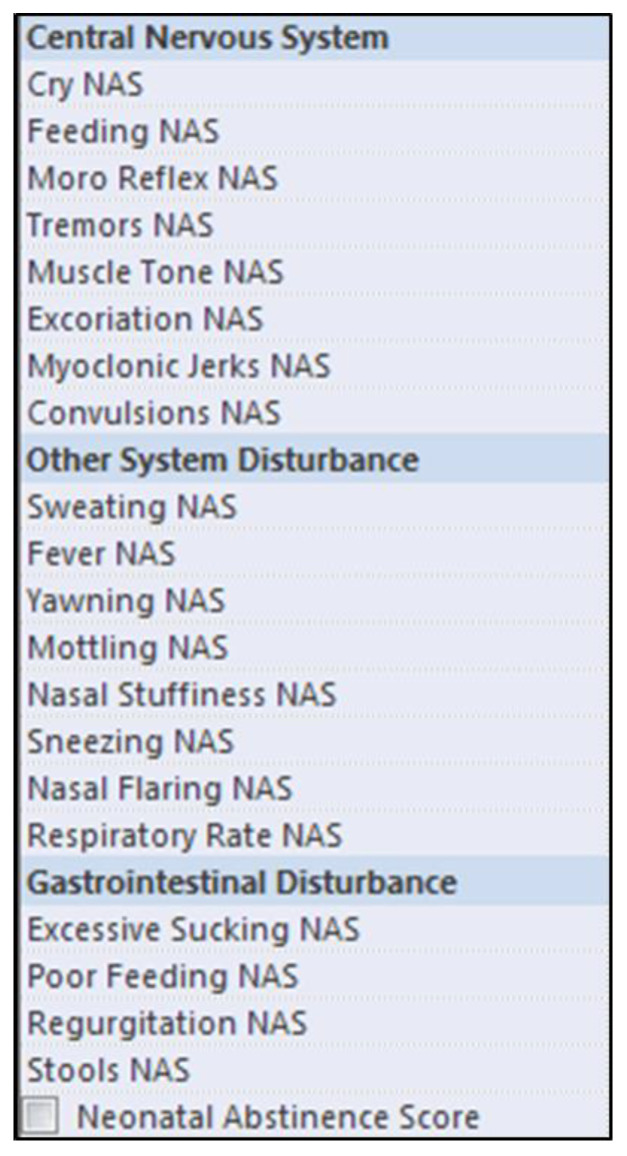
Modified Finnegan scoring (MFS). The current NAS scoring tool used at our NICU. Symptomatology is broadly categorized into 3 groups: central nervous system (CNS), other and gastrointestinal (GI).

The unit nursing protocol at the time included NAS scoring by nurses every 4 h using the MFS tool shown in Figure 1. When calculating total scores, each item can be worth 1 or more points depending on severity (e.g., sleep < 1 h after feeding scores as 3, whereas sleep < 2 h after feeding scores as 2). Per hospital policy, patients were started on pharmacotherapy with morphine following three consecutive scores ≥ 8 or two scores ≥ 12. Decisions to escalate or de-escalate the dosing of morphine were often based on the same cutoff values. Importantly, the prevalence of symptomatology in our study was arbitrarily defined by the presence of ≥3 symptoms listed under a symptom category at any single point in time during the patient’s NAS course. For example, if a patient was found to have excessive sucking, poor feeding and regurgitation during a single NAS scoring, they were considered to be prevalent in GI symptomatology. Subjects could be recorded as having prevalence in 1, 2 or all 3 symptom categories in this fashion.

### 2.2. Inclusion and Exclusion Criteria

Infants were included in our study if they were born between 2014 and 2020 and were given a diagnosis of NAS or an NAS-equivalent diagnosis in the Electronic Medical Record (EMR). NAS-equivalent diagnoses include “maternal history of substance use” or “infant affected by maternal substance use”. These synonymous diagnoses entries were included in our query to capture all the patients affected.

Two exclusion criteria were applied to our study population prior to data analysis. Firstly, patients who did not have documented Finnegan scores in EMR were excluded. Our institution receives patient transfers from non-pediatric facilities for the management of NAS. However, many of these previously diagnosed patients did not show clinical signs of NAS at our institution and hence were excluded. We also found several instances where patients were given a diagnosis of “maternal history of substance use” due to substances that are unlikely to cause NAS by themselves, such as Tetrahydrocannabinol (THC). Excluding patients who did not exhibit adequate concerning signs to be scored helped us avoid these patient populations.

The second exclusion criterion precluded significantly ill infants from this retrospective study. Patients with the following diagnoses during their hospital course were left out of this study: intraventricular hemorrhage (IVH), necrotizing enterocolitis (NEC), genetic abnormalities, surgical conditions/fasting status or respiratory failure requiring invasive support (i.e., intubation). These significant co-morbidities would naturally interfere with scoring of NAS and therefore skew our results. 

### 2.3. Study Outcomes

Primary: Prevalence of GI/CNS/Other symptomatology and maximum NAS scores measured with the Modified Finnegan Scoring (MFS) tool in preterm and term groups. Similar to previous studies measuring NAS outcomes, we elected to follow maximum NAS scores, as this measure is less impacted by scoring frequency or onset [12,13]. Scoring is often prompted by hunger cues, for which the timing can be inconsistent. This is especially the case in premature infants, who have their hunger cues suppressed early on in life via feeding tubes.

Secondary: Pharmacotherapy with morphine, which at our institution is considered as part of first-line treatment. Consideration was given to oral (PO) versus intravenous (IV) routes of morphine administration, and in the rare cases of IV morphine use in our subjects, a conversion dose ratio of 3:1 was used for PO: IV [14]. We also measured maximum morphine dose and duration of morphine therapy in days.

Other outcomes: Length of hospitalization (including days spent as inpatient after being transferred to the pediatric floor), use of second-line pharmacotherapy with phenobarbital, and neurodevelopmental outcome with Bayley Scales of Infant and Toddler Development (BSID-III) assessment. Maximum NAS scores and pharmacotherapy with morphine in infants with maternal breast milk (MBM)-inclusive diets versus formula-exclusive diets were also measured.

### 2.4. Statistical Analysis

Statistical analyses of the data were performed using GraphPad Prism software. Patient/maternal characteristics and outcomes were compared between preterm and term patient populations. Values were represented as means with 95% confidence intervals and medians with interquartile ranges (IQR) if appropriate. The Mann–Whitney U test was used to compare nonparametric data, while Fisher’s exact test and chi-square were used to compare proportions. Linear regression with an r^2^ value was performed to compare the correlation between maximum NAS scores and gestational age. *p*-values of ≤0.05 were considered to be significant.

## 3. Results

Our retrospective review yielded a total of 166 infants between 2014 and 2020, with 52 preterm and 114 term infants. Mean and standard deviations for the preterm and term groups were 35.1 (±1.2) and 39.1 (±1.1) weeks, respectively. Likewise, the average birth weight in the preterm group (2360 g ± 500 g) was significantly less than in the term group (3040 g ± 500 g).

### 3.1. Maternal Characteristics

In terms of maternal exposures, there were no significant differences found between the preterm and term groups (Table 1). The vast majority of drug exposures consisted of opioid use, which was present in 98% of cases equally in both groups. Less common maternal drug co-exposures included THC (25% of both groups), cocaine (25% versus 18% in mothers of term infants) and benzodiazepines (12% versus 14% in mothers of term infants). Anti-depressant use (i.e., SSRI or SNRI) was present in 15% of preterm cases and 23% of term cases. Tobacco and alcohol use rates were comparable between the two groups as well.

### 3.2. Infant Characteristics

The only significant difference, other than gestational age and birth weight, was the presence of respiratory conditions in preterm infants. This group more often had a comorbid respiratory condition, such as transient tachypnea of the newborn, pneumothorax or pneumonia (48% versus 15% in term infants). In contrast, the presence of a non-respiratory comorbidity (e.g., SGA status, hyperbilirubinemia, hypoglycemia, thrombocytopenia, suspected hepatitis C, seizures, etc.) was not significantly different across gestational ages. The proportion of term and preterm infants found to have a diagnosis of SGA or IUGR were comparable, at 20% versus 17%, respectively. Likewise, the proportion of infants who had breast milk incorporated into their diet was equivalent at 25%. 

### 3.3. NAS Outcomes

**Figure 2:**
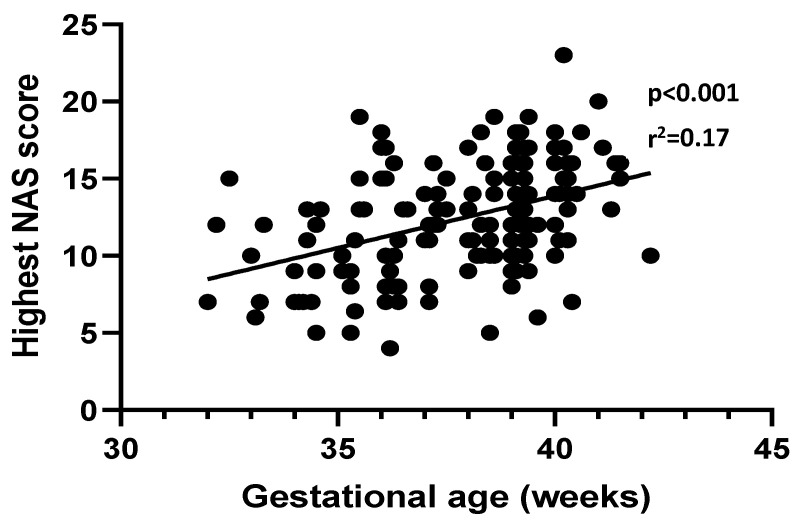
Maximum NAS scores versus gestational age shows a weak correlation with increasing MFS with gestational age.

The preterm group had a lower maximum NAS score than the term group (10 versus 13, respectively, *p* < 0.0001; see Table 2). Consequently, preterm infants less often required pharmacological therapy with morphine (52% versus 75%, *p* = 0.007). There was a significant, albeit weak, positive correlation overall between max NAS scores and gestational age (*p* < 0.001, r^2^ = 0.17; see Figure 2). Other markers of NAS severity were not found to be significantly different. Average days on morphine therapy were 12.6 versus 14 in the term group (*p* = 0.14). Median maximum oral morphine doses were also comparable between the two groups (0.04 mg/kg/dose versus 0.05 mg/kg/dose in the term group, *p* = 0.49). Only two term and zero preterm infants required the use of second-line pharmacological therapy with phenobarbital for NAS management (*p* = 1.0). The median length of hospitalization was 15 days for preterm infants and 13 for term infants.

### 3.4. Symptomatology

Preterm infants with NAS less often experienced GI symptoms (i.e., excessive sucking, poor feeding, reflux and loose stools) than term infants with NAS (25% versus 44%, *p* = 0.02). The presence of neurological symptomatology and other symptomatology categories were similar between the study groups (Table 2).

### 3.5. Feeding Regimen

Regardless of gestational age, patients with MBM incorporated into their diet less often required treatment with morphine (50% versus 71%, *p* = 0.01). There was no significant difference in median max NAS scores between infants with MBM and those who were exclusively formula fed (Table 3).

## 4. Discussion

Our understanding of the withdrawal process in neonates has lagged behind our current appreciation of prematurity and its associated complications. In neonatal–perinatal practice, many exceptions are made for managing and treating disorders in premature infants, often tailored towards their more fragile and unpredictable state. However, NAS continues to be approached analogously regardless of gestational age. 

The data presented here demonstrate that premature infants are less likely to present with GI symptoms during their course of NAS. Simply put, this could also explain the lower NAS scores and need for morphine therapy in preterm infants found in our study. It is important to note how the MFS tool could be influenced by a difference in symptomatology. The version of the MFS tool employed at our institution only has four items in the GI category that infants can score for. For comparison, the CNS and Other scoring categories have eight items each. It stands to reason that premature infants may be underscoring relative to term infants because the contribution of all three categories on the scoring tool are not equivalent. Likewise, significant neurological development occurs in the third trimester, and premature infants may, consequently, score lower in the CNS category [15,16]. Although this was not a focus of our study, previous literature has even found that preterm infants who met the threshold for beginning pharmacological therapy were less likely to be treated as such when compared to their term counterparts [14].

Furthermore, the MFS tool makes no consideration for the known tendencies of prematurity. Infants born at earlier gestational ages are more likely to have decreased orientation, decreased tolerance for handling, decreased self-regulation, decreased reflexes, changes in tone, increased stress and excitability [17]. Poor feeding is another common challenge for premature infants, since they do not develop their suck and swallow coordination until approximately week 32 to 34 of life. They are also prone to increased reflux due to decreased lower esophageal tone and the presence of a nasogastric tube [18]. Nasal flaring and tachypnea would also be expected more often in premature infants due to their lack of surfactant development and their predisposition for respiratory distress syndrome. 

The current stance of the AAP on NAS scoring is that the organization does not support any scoring system over another, as there is no conclusive evidence to suggest superiority [11]. Instead, it stresses the importance of consistency in protocol and physician adherence. Our findings here contend that a single universal scoring tool that ignores gestational age may be inappropriate for the management of NAS. There are several previously proposed explanations for why premature infants undergo a less severe withdrawal course. For example, immature opioid receptors and slower placental transfer rates may cause premature infants to be less impacted by maternal opioid use [7]. Premature infants are inherently exposed in utero for a shorter duration and mothers tend to require increasing amounts of opioids as their pregnancies progress. As previously mentioned, significant neurological development occurs in the third trimester, and infants born early could be spared from opioid exposure during this critical time.

The duration of morphine therapy was considered to be superior to length of stay for approximating the severity of NAS, as it is rare for NICU infants to have their hospital course complicated by NAS alone. In our study population specifically, 81% of preterm and 64% of term infants had other major illnesses. The average duration of morphine therapy was not found to be significantly different here, but a possible explanation is that providers may hesitate to wean morphine on days where a patient is unstable due to other medical conditions. Such a situation is more likely to occur in preterm infants and, therefore, prolong their morphine course, regardless of NAS scores. Significantly, the upper limit of days on morphine was 57 for term infants and 27 for preterm infants. This difference is both anecdotally familiar at our institution and well described in previous literature [1,7,19]. 

Maximum utilized morphine dose was also not found to be significant in our study. The study conducted by Ruwanpathirana et al. did report lower maximum morphine doses in preterm infants [13]. However, we had a higher incidence of respiratory distress in our preterm cohort (48% as compared to 34%), which could contribute to more clinical signs mimicking NAS symptoms. Use of a second-line pharmacological agent with phenobarbital was also not significantly different with gestational age. Our study attempted to track neurodevelopmental sequela, as well, to make a comparison across gestational ages. Unfortunately, a combination of poor follow-up and a lack of EMR documentation precluded the collection of such data.

Our study is limited in a few ways. As a retrospective chart review, it can be affected by ascertainment bias, incorrect documentation and missed cases. The data referenced here were also collected from a single institution, which may not be representative of other populations. Due to limitations in EMR documentation, specific maternal opioid dosages used could not be determined, although we would expect them to be randomized between our study groups. Finally, stable infants with NAS at our institution are often transferred to the inpatient pediatric floor for further management. Term infants were accordingly more likely to be transferred out of the ICU and, therefore, be managed by non-ICU physicians and nurses for a portion of their hospital stay. 

## 5. Conclusions

In summary, our data suggest that preterm infants with NAS are less likely than their term counterparts to have higher NAS scores, and thus requiring pharmacological treatment, when measured with conventional scoring methodologies, such as the MFS tool. There is also a difference in NAS symptomatology experienced by infants of varying gestational age. Our study further supports the inclusion of MBM in the diets of infants with NAS, as it appears to be associated with a reduced need for pharmacological treatment. There is a need for newer NAS assessments that can overcome the existing challenges of traditional scoring systems, and which are validated in preterm infants. 

## Figures and Tables

**Table 1:** Maternal and Infant Characteristics.

Demographics	Preterm (52)	Term (114)
Gestational Age (Weeks)	35.1 ± 1.2	39.1 ± 1.1 ^1^
Average Birth Weight (kg)	2.36 ± 0.5	3.04 ± 0.5 ^1^
Diet Includes Breast Milk	13 (25%)	29 (25%)
SGA/IUGR ^2^	9 (17%)	23 (20%)
Respiratory Condition	17 (48%)	17 (15%) ^1^
Other Condition	42 (81%)	73 (64%)
Drug Exposures	Opioids 51 (98%)/Cocaine 13 (25%)/THC 13 (25%)	Opioids 112 (98%)/Cocaine 20 (18%)/THC 29 (25%)
SSRI/SNRI	8 (15%)	26 (23%)
Tobacco	41 (79%)	80 (70%)

^1^*p*-value is ≤ 0.05. ^2^ SGA is defined as < 10th percentile for age per Fenton’s growth chart.

**Table 2:** NAS Outcomes and Symptomatology.

Outcomes	Preterm (52)	Term (114)	*p*-Value
Maximum NAS Score (Median, IQR)	10 (7–13)	13 (11–16)	<0.0001 ^2^
% Requiring Morphine	27 (52%)	85 (75%)	0.007 ^2^
Average/Range of Morphine Duration (Days)	12.6 (13.1 ^a^)/1–27	14 (14.8 ^a^)/1–57	0.14
Max PO Morphine Dose (Median, IQR)	0.04 mg/kg/dose (0.04–0.05)	0.05 mg/kg/dose (0.04–0.05)	0.49
Use of 2nd line agent	0	2 (0.018%)	1.0
(Phenobarbital)			
Length of Hospitalization (median, IQR)	15 (11–20)	13 (7–19)	ns
Neurological Symptomatology	51 (98%)	114 (100%)	
GI Symptomatology	13 (25%)	50 (44%)	0.02 ^1^

^1^*p* ≤ 0.05, ^2^ *p* ≤ 0.01, ns *p* = non-significant. ^a^ Excludes subjects receiving 1 day of pharmacotherapy.

**Table 3:** Effect of Feeds on NAS Scores and Need for Pharmacotherapy.

Outcomes	Inclusive of MBM (42)	Exclusively Formula (128)	*p*-Value
Maximum NAS Scores (Median, IQR)	11.5 (9–14.25)	13 (10–15)	0.08
Required Morphine	21 (50%)	91 (71%)	0.01 ^1^

^1^*p*-value is ≤ 0.05.

## Data Availability

The data presented in this study are available within this article.
